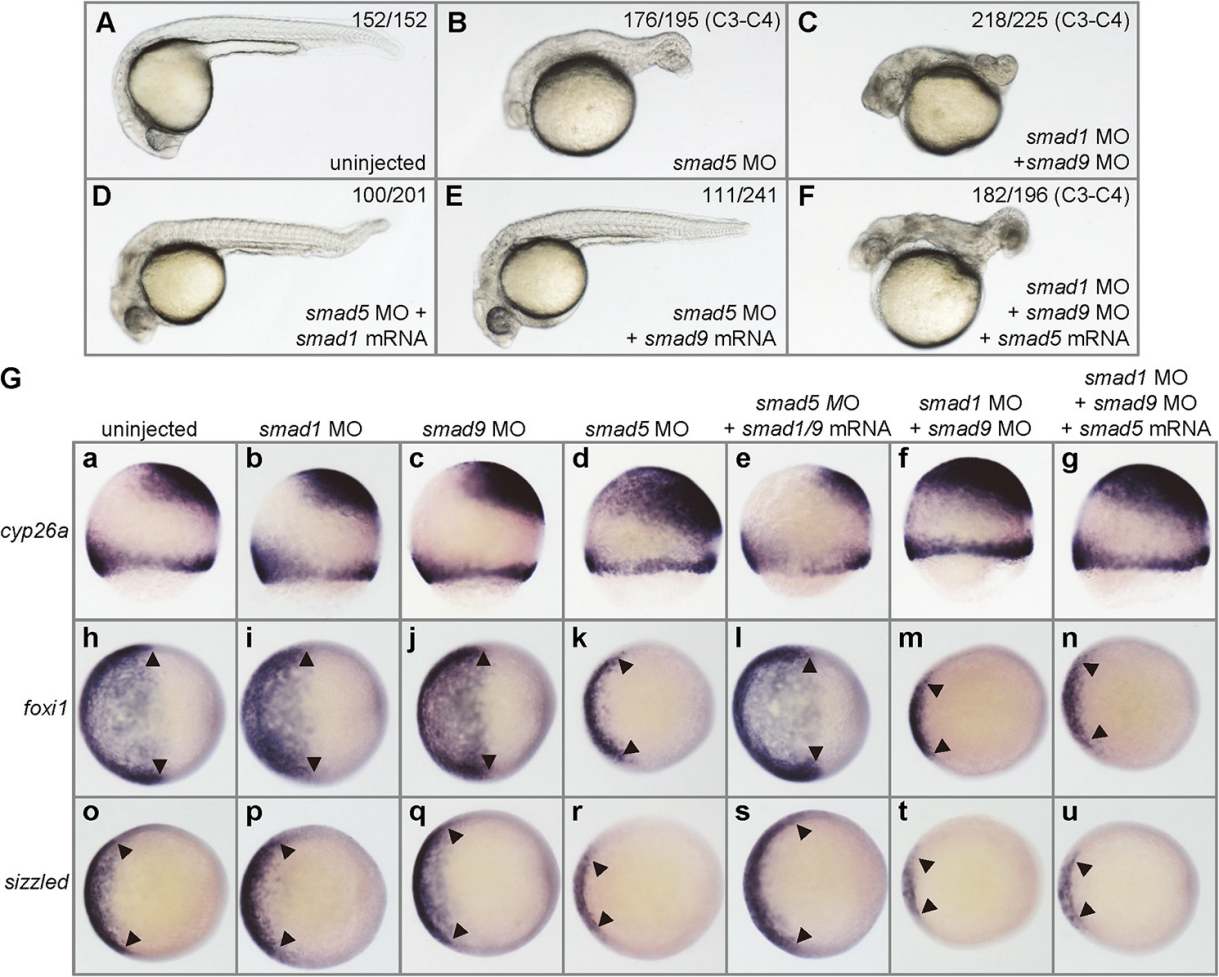# Correction: Transcriptional factors Smad1 and Smad9 act redundantly to mediate zebrafish ventral specification downstream of Smad5.

**DOI:** 10.1074/jbc.AAC120.016989

**Published:** 2021-01-13

**Authors:** Chang-Yong Wei, Hou-Peng Wang, Zuo-Yan Zhu, Yong-Hua Sun

VOLUME 289 (2014) PAGES 6604–6618

In Fig. 4*G*, in the *foxi1 panel*, the images in Fig. 4*G*, *i* and *l*, corresponding to “smad1 MO” and “smad5 MO + samd1/9 mRNA” samples, respectively, were inadvertently reused during figure preparation. This error has now been corrected using images pertaining to each treatment and sample. This correction does not affect the results or conclusions of the work.

**Figure 4G:**